# Multilocus Sequence Typing Reveals both Shared and Unique Genotypes of *Cryptococcus neoformans* in Jiangxi Province, China

**DOI:** 10.1038/s41598-018-20054-4

**Published:** 2018-01-24

**Authors:** Yan-Hui Chen, Feng Yu, Ze-Yuan Bian, Jian-Ming Hong, Nan Zhang, Qiao-Shi Zhong, Ya-Ping Hang, Jianping Xu, Long-Hua Hu

**Affiliations:** 1 Jiangxi Provincial Key Laboratory of Medicine, Clinical Laboratory of the Second Affiliated Hospital of Nanchang University, Nanchang, Jiangxi China; 2 Clinical Laboratory of Nanchang Chest Hospital, Nanchang, Jiangxi China; 3 Clinical Laboratory of the Ninth Hospital of Nanchang, Jiangxi, China; 4 Institute of Infectious Disease Research, Michael G. DeGroote School of Medicine, and Department of Biology, McMaster University, Hamilton, Ontario L8S 4K1 Canada

## Abstract

Cryptococcosis is a globally distributed infectious fungal disease. However, much remains unknown about its molecular epidemiology in many parts of the world. In this study, we analyzed 86 clinical *Cryptococcus neoformans* isolates from 14 regions in Jiangxi Province in south central China. Each isolate was from a different patient and 35 of the 86 (40.7%) patients were infected with HIV. All strains belonged to serotype A and mating type *α* (MATα). Genotyping based on DNA sequences at seven nuclear loci revealed eight sequence types (STs) among the 86 isolates, including two novel STs that have not been reported from other parts of the world. ST5 was the dominant genotype and our comparative analyses showed that these genotypes in Jiangxi likely originated by dispersal from other regions within and outside of China and/or mutations from another genotype within Jiangxi. Though none of the isolates was resistant to the five tested antifungal drugs (flucytosine, amphotericin B, fluconazole, itraconazole, and voriconazole), obvious differences in their minimum inhibitory concentrations were observed, even among isolates of the same ST. Our results suggest that continuous monitoring should be conducted to understand the changing dynamics of *C. neoformans* in this and other regions.

## Introduction

*Cryptococcus* is a genus of basidiomycetous fungi^1^. Two species in this genus, *Cryptococcus neoformans* and *Cryptococcus gattii*, are major pathogens of humans and other animals and can cause a diversity of diseases collectively called cryptococcosis^1^. Each year, approximately 960,000 new cases of cryptococcal meningitis occur in predominantly HIV-infected patients. In regions without access to antiviral and antifungal treatments, ~60% of those with cryptococcal meningitis die soon after infection^2^. While most of the deadly infections are in Sub-Sahara Africa, several other regions have also reported increasing cases, including China^3^. According to the latest review, 8,769 cases of cryptococcal infections were reported in China from 1985 to 2010, many of which were in HIV-negative hosts^3^. For successful control and prevention of cryptococcosis, it’s critical to understand the molecular epidemiology of cryptococcal infections in these emerging regions.

Both *C. neoformans* and *C. gattii* have broad geographic distributions and they can grow in a diversity of environments. For example, they are commonly found in soil, pigeon droppings, and debris of trees such as *Eucalyptus camaldulensis*^4–6^. Jiangxi Province in south central China has a subtropical environment ideally suited for the growth and reproduction of both *C. neoformans* and *C. gattii*, and contains a large number of *E. camaldulensis* trees^6^. However, little is known about the populations of these two species in Jiangxi Province. For example, only five clinical strains of *C. neoformans* from Jiangxi have been reported in previous reports^6^.

To understand the genotypes and molecular epidemiology of cryptococcal infections in Jiangxi Province, here we analyzed 86 cases of clinical cryptococcosis in patients from different regions of Jiangxi. The isolates were first identified based on the Matrix-assisted laser desorption/ionization time-of flight mass spectrometry (MALDI-TOF MS)^7,8^. The isolates were then analyzed for their mating types, multilocus genotypes derived based on sequencing at seven nuclear loci, and susceptibilities to five antifungal drugs.

The taxonomy and systematics of the human pathogenic *Cryptococcus* have undergone multiple revisions and a broad consensus is still not available. In this study, we follow the commonly used approach that sub-divide the human pathogenic *Cryptococcus* into two species complexes, the *C. neoformans* species complex (CNSC) and the *C. gattii* species complex (CGSC). CNSC includes three serotypes (serotypes A, D, and AD) and four molecular types (VNI, VNB/VNII, VNIII and VNIV). This species complex is mainly responsible for cryptococcal infections in AIDS patients. CGSC has two serotypes (serotypes B and C), four molecular types (VGI, VGII, VGIII and VGIV), and is geographically relatively limited and more commonly diagnosed in immuno-competent than immuno-compromised individuals^9–12^. Several molecular techniques have been employed for identifying the molecular types and/or genotypes for strains in these two species complexes, including polymerase chain reaction (PCR) fingerprinting, pulsed-field gel electrophoresis (PFGE), amplified fragment length polymorphism (AFLP), multilocus microsatellite typing (MLMT), repetitive-sequence-based PCR, restriction fragment length polymorphism of the *URA5* gene (*URA5*-RFLP), and multilocus sequence typing (MLST)^13^. Among these methods, MLST has become the preferred method by the International Society for Human and Animal Mycology (ISHAM)^11^. The objectives of this study are to analyze the genotypes and antifungal drug susceptibilities of isolates causing cryptococcosis in Jiangxi Province and to compare their genotypes with those from other regions.

## Materials and Methods

### **C**ryptococcal Isolates

In this study, 86 isolates of *C. neoformans* were obtained from patients hospitalized in Jiangxi hospitals from January 2016 to November 2017. These patients came from all major regions of the Province, spanning ~600 km from the south (Ganzhou) to north (Jiujiang) and ~500 km from east (Leping) to west (Pingxiang). The detailed information about each of the samples is presented in Table 1. Request for the clinical isolates and patient information followed institutional guidelines of Nanchang University. The isolates were stored in skim milk at −80 °C until use and were maintained on SDA (Sabouraud Dextrose Agar, 1% yeast extract, 2% peptone, 2% glucose, 1.8% agar) medium at 25 °C during this study for genotyping and MIC testing.

**Table 1:** Information of the 86 clinical isolates of *Cryptococcus neoformans* in Jiangxi Province, China.

Isolate	Location	Sex	Age	Specimen	Underlying condition	ST
CN9	Dexing	F	39	CSF	HIV(+)	5
CN6	Dexing	M	49	CSF	Immunocompetent	5
CN1	Fengcheng	F	74	Blood	Systemic lupus erythematosus	5
CN8	Fuzhou	F	62	Blood	Anca - associated vasculitis	5
CN51	Fuzhou	F	63	CSF	Chronic hepatitis	5
CN12	Fuzhou	M	37	Blood	HIV(+)	5
CN56	Fuzhou	M	31	Blood	HIV(+)	5
CN2	Fuzhou	M	33	CSF	HIV(+)	5
CN26	Fuzhou	M	28	CSF	Immunocompetent	139
CN69	Fuzhou	M	48	CSF	Nephrotic syndrome	5
CN45	Fuzhou	M	47	Blood	Unknown	5
CN76	Fuzhou	M	68	CSF	Chronic hepatitis	359
CN28	Ganzhou	M	18	Blood	Immunocompetent	5
CN41	Ganzhou	M	64	CSF	Malignant lymphoma	31
CN63	Ganzhou	F	50	CSF	Unknown	5
CN88	Ganzhou	F	66	CSF	Emphysema	5
CN92	Ganzhou	M	57	CSF	Chronic hepatitis	5
CN37	Gaoan	F	43	CSF	Unknown	5
CN14	Ji’an	F	48	CSF	HIV(+)	5
CN49	Ji’an	M	44	Blood	HIV(+)	5
CN61	Ji’an	M	22	CSF	HIV(+)	5
CN21	Ji’an	M	43	Sputum	Unknown	319
CN50	Ji’an	F	36	CSF	Unknown	5
CN53	Ji’an	F	61	Blood	Unknown	5
CN73	Ji’an	F	28	CSF	Kidney transplant	5
CN74	Ji’an	M	67	CSF	Tuberculosis	5
CN79	Ji’an	F	33	CSF	HIV(+)	5
CN94	Ji’an	F	51	Blood	SLE	359
CN36	JiuJiang	F	67	CSF	Diabetes mellitus	5
CN31	Jiujiang	M	39	Blood	HIV(+)	5
CN48	Jiujiang	F	69	CSF	HIV(+)	5
CN65	Jiujiang	M	37	Blood	HIV(+)	5
CN68	Jiujiang	M	49	CSF	Immunocompetent	5
CN64	Jiujiang	M	65	CSF	Unknown	5
CN86	Jiujiang	M	25	CSF	HIV(+)	5
CN87	Jiujiang	M	62	CSF	Myasthenia gravis	5
CN27	Leping	M	53	CSF	Chronic hepatitis	5
CN19	Leping	M	42	CSF	HIV(+)	5
CN44	Leping	M	38	CSF	HIV(+)	5
CN3	Leping	M	42	CSF	Immunocompetent	186
CN10	Leping	M	44	Blood	Malignant lymphoma	5
CN34	Nanchang	M	38	CSF	Brain trauma	5
CN7	Nanchang	M	70	Blood	Chronic hepatitis	5
CN11	Nanchang	M	23	Blood	HIV(+)	5
CN22	Nanchang	M	49	CSF	HIV(+)	5
CN23	Nanchang	M	33	CSF	HIV(+)	5
CN35	Nanchang	M	40	CSF	HIV(+)	5
CN58	Nanchang	M	26	CSF	HIV(+) + tuberculosis	5
CN18	Nanchang	M	76	CSF	Immunocompetent	5
CN24	Nanchang	M	20	CSF	Immunocompetent	5
CN13	Nanchang	M	52	CSF	Kidney transplant	359
CN39	Nanchang	F	33	CSF	Systemic lupus erythematosus	5
CN16	Nanchang	M	38	Marrow	Tuberculosis	5
CN57	Nanchang	F	74	CSF	Unknown	5
CN71	Nanchang	M	41	CSF	HIV(+)	5
CN81	Nanchang	M	48	CSF	Malignant tumor	5
CN85	Nanchang	M	79	CSF	Chronic steroid usage	359
CN93	Nanchang	M	51	CSF	Silicosis	5
CN46	Pingxiang	M	50	CSF	Unknown	5
CN29	Shangrao	M	65	Blood	Chronic hepatitis	5
CN30	Shangrao	F	43	Blood	HIV(+)	32
CN32	Shangrao	M	41	Blood	HIV(+)	5
CN33	Shangrao	F	38	Blood	HIV(+)	5
CN42	Shangrao	M	27	CSF	HIV(+)	5
CN52	Shangrao	F	55	CSF	HIV(+)	5
CN54	Shangrao	M	45	CSF	HIV(+)	5
CN67	Shangrao	M	55	CSF	HIV(+)	5
CN70	Shangrao	M	26	CSF	HIV(+)	5
CN40	Shangrao	M	29	CSF	HIV(+) + tuberculosis	5
CN4	Shangrao	M	53	CSF	Immunocompetent	5
CN15	Shangrao	F	50	CSF	Unknown	5
CN75	Shangrao	F	61	CSF	Immunocompetent	5
CN80	Shangrao	F	55	CSF	HIV(+)	5
CN84	Shangrao	M	62	CSF	Immunocompetent	5
CN66	Xinyu	F	4	CSF	Anaphylactoid purpura	5
CN91	Xinyu	F	27	CSF	HIV(+)	5
CN17	Yichun	M	50	CSF	Cerebral infarction	5
CN25	Yichun	M	52	Hydrothorax	Drug taking and diabetes mellitus	226
CN43	Yichun	M	31	CSF	HIV(+)	5
CN38	Yichun	M	51	CSF	Nephrotic syndrome	5
CN60	Yichun	M	67	CSF	Unknown	5
CN82	Yichun	M	17	CSF	Nephrotic syndrome	186
CN20	Yingtan	M	26	CSF	HIV(+)	5
CN55	Yingtan	M	61	CSF	HIV(+)	5
CN83	Yingtan	M	40	CSF	tuberculosis	186
CN90	Yingtan	F	49	CSF	HIV(+)	186

### DNA Extraction

Genomic DNA was extracted from each isolate following the protocol described by Alessandro *et al*.^14^, with slight modifications. Briefly, cells were incubated on SDA agar containing 0.5 M NaCl at 30 °C overnight. Protoplasts were generated by incubating cells in 2 ml of urea buffer (8 M urea, 0.5 M NaCl, 20 mM Tris, 20 mM EDTA, 2% SDS (Sigma, USA), pH 8.0) for 3–4 h at room temperature. The protoplasts were collected by centrifugation and vortexed in 400 μl lysis buffer (1% w/v SDS in TE, pH 7.5). After vortexing, 400 μl of phenol-chloroform (1:1, pH 8.0) and 400 μl of 2-μm acid-washed glass beads were added and further vortexed. The mixes were centrifuged and the extracted DNA was washed with 100% ethanol, re-suspended in 100 μl TE, and then stored at −20 °C.

### Identification of Species, Lineages, and Mating Types

To confirm that the isolates all belonged to the human pathogenic *Cryptococcus* species complexes, we used the MALDI-TOF MS (BioMerieux, Marcy L’Etoile, France), following the protocols described in Mctaggart *et al*. using the on-plate protein extraction method^8^. Briefly, isolates were first cultured on SDA and incubated at 30 °C for 24 h. One single colony of each isolate was smeared onto each MALDI-TOF MS analysis plate, and the proteome of each isolate was extracted via 0.5 μl formic acid and 1.0 μl matrix liquid. The protein profile was automatically generated for those proteins with molecular weights ranging from 2 to 20 kDa. *Escherichia coli* ATCC8739 was used as a quality control.

To identify whether the isolates belonged to either the *C. neoformans* species complex or the *C. gattii* species complex, we plated all isolates on L-canavanine-glycine-bromothymol blue agar^15^, followed by sequencing of all isolates at the *SOD1* gene^16^. The sequences were then compared with those of five strains representing the known molecular types of *C. neoformans*: WM148 (serotype A, VNI), WM 626 (serotype A, VNII), Bt63 (serotype A, Botswana), WM 628 (serotype D, VNIII), and WM629 (serotype AD, VNIV) as well as those in the GenBank and the ISHAM MLST database. Furthermore, the standard strains H99 (serotype A, MATα), JEC21 (serotype D, MATα), and JEC20 (serotype D, MATa) were used as references to determine the serotype and mating type for each of the isolates, following the methods described by Yan *et al*. using serotype and mating type-specific primers at the *STE20* gene for PCR^17^. These primers target the *STE20Aa*, *STE20Aα*, *STE20Da* and *STE20Dα* alleles. After amplification, all the PCR products were electrophoresed on 1% agarose gels in 0.5xTBE buffer at 100 V for 60 min and then visualized under UV light by comparison with their reference strains.

### MLST Analysis

Aside from obtaining the *SOD1* gene sequence for each of the isolates for species identification, we also obtained the sequences at six other genes as suggested by the ISHAM consensus MLST scheme for *C. neoformans* and *C. gattii*^11^. Briefly, these six DNA fragments were located in the following genes *CAP59, GPD1, IGS1, LAC1, PLB1*, and *URA5*. Primers and PCR conditions followed that described in Hiremath *et al*.^18^. All sequences were submitted to the National Center for Biotechnology Information (NCBI) database to acquire GenBank accession numbers and the *C. neoformans*/*C. gattii* species complex database (http://mlst.mycologylab.com) to obtain sequence type (ST) numbers.

### Phylogenetic Analysis

Phylogenetic analysis of the concatenated sequences of seven MLST loci was performed using MEGA version 7.0 software^19^. A phylogenetic tree was produced by the Neighbor-Joining algorithm using alignments of the concatenated sequences at the seven gene loci from our isolates and two reference strains H99 and WM148.

### Comparison with Other Geographic Populations from China

To investigate the potential genetic differences between the Jiangxi population of *C. neoformans* and those from other geographic regions in China, we extracted all the published genotype information for all the Chinese isolates at the seven sequenced loci from the *Cryptococcus* MLST database (http://mlst.mycologylab.com). These Chinese populations were then analyzed using the GenAlEx software (version 6.5)^20^. Two analyses were performed. In the first, the overall genetic variation was partitioned into within and between geographic populations through AMOVA. In the second, the genetic differences between all pairwise geographic populations were analyzed. All regional populations with a sample size of greater than five isolates were included in the above analyses. Statistical significance of the observed genetic differences was determined by 1000 permutations using the GenAlEx software^20^.

### Antifungal Susceptibility Testing

The *in vitro* antifungal susceptibility testing of all 86 isolates of amphotericin B (AMB), flucytosine (5FC), fluconazole (FLU), voriconazole (VOR), itraconazole (ITR) was performed using the ATB^TM^ FUNGUS-3 kit (BioMerieux, Marcy L’Etoile, France). The minimal inhibitory concentrations (MIC) were determined following instructions provided by the User’s Manual. *Candida krusei* ATCC6258 and *Candida parapsilosis* ATCC22019 were used as reference quality controls.

The obtained MIC values were compared to those recommended breakpoints to determine whether the strains were susceptible or resistant to specific antifungal drugs. The MIC breakpoints for fluconazole and flucytosine were ≥16 μg/ml and ≥32 μg/ml respectively as suggested based on the User’s Manual of ATB^TM^ FUNGUS-3. For amphotericin B, we followed the resistance breakpoint of ≥2 μg/ml as suggested by CLSI document M27-A3^21^ and Nguyen *et al*.^22^. At present, there are no consensus interpretive breakpoints of ITR and VOR based on the ATB^TM^ FUNGUS-3 system for *C. neoformans*. Here we follow previous studies and used a MIC ≥ 1 μg/ml as the resistance breakpoint for both ITR and VOR^23,24^.

### Data availability statement

All the data described in this manuscript are presented in the paper (for genotype information and MIC values of all 86 isolates) as well as deposited in the publicly accessible database (http://mlst.mycologylab.com) for all nucleotide sequences.

### Statements on study approvals

We confirm that all methods used in this study were carried out in accordance with relevant guidelines and regulations. In addition, all experimental protocols were approved by Nanchang University and that informed consent was obtained from all subjects for the *Cryptococcus neoformans* isolates analyzed in this study.

## Results

### Demographic Data of the Clinical Isolates

In total, 86 clinical isolates of *C. neoformans* were obtained from patients in 14 cities/counties distributed across Jiangxi Province (Table 1). Each of these isolates was from a different patient. Of the 86 isolates, 60 originated from male patients and 26 from female patients. The age distribution of these 86 cases ranged from 4 to 79 years, and the numbers from each age group were as follows: four (≤20 years), 11 (21–30 years), 17 (31–40 years), 22 (41–50 years), 11 (51–60 years), 17 (61–70 years), and four (>70 years). A majority of these isolates were obtained from cerebrospinal fluid (n = 65; 75.5%), followed by blood (n = 18; 20.9%), and one each from bone marrow, sputum and hydrothorax (n = 1 each; 1.2%). Of these 86 isolates, 35 (40.7%) were from HIV-positive individuals, 40 from HIV-negative hosts, and eleven from individuals of unknown disease status. The majority (31) of the 40 HIV-negative hosts (31/86 total hosts, 36.0%) had deficient or suppressed immune systems associated with cancer or liver disease treatments or chronic steroid usage. Only nine hosts (10.5%) had no known risk factors for cryptococcosis (Table 1).

### Serotype, Mating Types, and MLST Results

All 86 clinical isolates were identified as *C. neoformans* serotype A, molecular type VNI, and mating type α. MLST analysis divided the 86 isolates into eight sequence types (STs), including 73 isolates of ST5 (84.9%). Of the remaining 7 STs, two (ST186 and ST359) were represented by four isolates each while the remaining five (ST31, ST32, ST139, ST226, and ST319) were represented by one isolate each. The multilocus sequence types of all 86 strains are presented in Table 1. The 73 isolates with the ST5 multilocus genotype came from all sampled regions in Jiangxi Province. Similarly, the remaining two STs, ST186 and ST359, represented by four isolates each were also distributed broadly, in three regions each. The remaining five isolates each with a different multilocus genotype came from different regions of Jiangxi, including Shangrao (ST32) in the northeast; Fuzhou (ST139) and Ji’an (ST319) in the center; Yichun (ST226) in the west; and Ganzhou (ST31) in the south.

The allelic assignments of our individual gene sequences in the MLST database for each of the eight multilocus sequence types are presented in Table 2. Table 2 also shows the distributions of the individual alleles in the Jiangxi population among all the known sequence types in the MLST database. In total, 18 alleles were found at the seven loci in Jiangxi. Two loci, *SOD1* and *URA5* were monomorphic in Jiangxi and their alleles (#1 at both loci) were distributed broadly in many other STs within and outside of China. The remaining five loci were polymorphic, with allele numbers ranging from two (*CAP59*) to six (*GPD1*). At each of the remaining five loci, the Jiangxi population shared alleles with a diversity of known STs from other geographic regions (Tables 2 and 3). Among these 18 alleles at the seven loci, none was specific to Jiangxi and all have been found elsewhere (Tables 2 and 3).

**Table 2:** Allelic assignments of the eight multilocus sequence types found in this study.

ST	*CAP59* ^1^	*GPD1* ^2^	*IGS1* ^3^	*LAC1* ^4^	*PLB1* ^5^	*SOD1* ^6^	*URA5* ^7^	VN^8^
ST5	1	3	1	5	2	1	1	1
ST31	1	1	10	3	2	1	1	1
ST32	1	1	10	3	4	1	1	1
ST139	1	6	22	18	4	1	1	1
ST186	1	26	1	5	2	1	1	1
ST226	7	3	1	5	2	1	1	1
ST319	1	23	10	18	4	1	1	1
ST359	1	25	1	5	2	1	1	1

^1^*CAP59:* Allele #1 has been found in 139 known STs; allele #7 in 55 STs.

^2^*GPD1*: Allele #1 has been found in 106 known STs; allele #3 in 32 STs; allele #6 in 6 STs; allele ^#^23 in 20 STs; allele #25 in one ST (i.e. ST359); and allele #26 in one ST (i.e. ST186).

^3^*IGS1*: Allele #1 has been found in 124 known STs; allele #10 in 33 STs; allele #22 in one ST (ST139).

^4^*LAC1*: Allele #3 has been found in 65 known STs; allele #5 in 31 STs; and allele 18 in 39 STs.

^5^*PLB1*: Allele #2 has been found in 68 known STs; allele #4 in 71 STs.

^6^*SOD1*: allele #1 has been found in 183 STs.

^7^*URA5*: allele #1 has been found in 91 STs.

^8^VN: These eight STs found in Jiangxi are among 487 total STs in the VNI molecular type in the MLST database accessed on September 16, 2017.

**Table 3:** Summary distributions of the eight sequence types identified in Jiangxi Province in other parts of the world.

ST	Geographic location			Percentage of the population
ST5	Asia	China	Beijing	30.4% (34/112)^13,34^
			Shanghai	72.7% (16/22)^33^
			Sichuan Province	89.5% (119/133)^35,39^
			Guangdong Province	87.1% (27/31)^33^
			Henan Province	93.3% (14/15)^34^
			Heilongjiang Province	76.0% (19/25)^39^
			Liaoning Province	83.3% (10/12)^39^
			**Jiangxi Province**	**84.9% (73/86)**
			Hong Kong	85.7% (12/14)^33^
		Japan		61.4% (51/83)^30,33^
		Thailand		13.8% (41/297)^32,33,40^
		South Korea		56.2% (9/16)^37^
		Vietnam		47.8% (65/136)^41^
	United States of America			28.7% (58/202)^33,42,43^
	Europe			11.3% (8/71)^33,42,44^
	Brazil			2.1% (3/144)^42^
	South Africa			12% (28/230)^33,42,45^
ST31	Asia	China	Beijing	49.1% (55/112)^13,34^
			Hebei Province	8.6% (3/35)^39^
			Henan Province	6.7% (1/15)^34^
			Heilongjiang Province	4.0% (1/25)^39^
			Liaoning Province	8.3% (1/12)^39^
			Sichuan Province	7.5% (10/133)^35,39^
			**Jiangxi Province**	**1.2% (1/86)**
		Japan		1.2% (1/83)^33^
		Thailand		2.0% (6/297)^32,33^
		India		11.5% (7/61)^33^
	South Africa			0.4% (1/230)^45^
	Brazil			2.1% (3/144)^42^
ST32	Japan			1.2% (1/83)^30^
	United States of America			0.5% (1/202)^43^
	South Africa			8.3% (16/230)^33,45^
	Europe			1.4% (1/71)^42^
	Brazil			2.1% (3/144)^42^
	Vietnam			5.1% (7/136)^41^
	Thailand			0.3% (1/297)^40^
	**Jiangxi Province**			**1.2% (1/86)**
ST186	Shanghai			4.5% (1/22)^33^
	**Jiangxi Province**			**4.7% (4/86)**
ST139	Africa			Unknown frequency^33^
	**Jiangxi Province**			**1.2% (1/86)**
ST359	Zhejiang Province			9.1%(1/11)^35^
	Hebei Province			2.9%(1/35)^35^
	**Jiangxi Province**			**4.7% (4/86)**
ST319	**Novel, Jiangxi Province**			**1.2% (1/86)**
ST226	**Novel, Jiangxi Province**			**1.2% (1/86)**

Among the eight multilocus STs in our Jiangxi population of *C. neoformans*, six (ST5, ST31, ST32, ST139, ST186, and ST359) have been reported previously from other geographic areas (Table 3). The remaining two genotypes (ST226 and ST319) have only been found in our study population. The geographic distributions of these eight STs are shown in Table 3. Of the six shared STs between Jiangxi and other regions, three (ST5, ST31, and ST32) have been found in multiple continents/countries. For example, ST5 has been reported from the US, Europe, Brazil, South Africa, and several countries in eastern and southeastern Asia. The high prevalence of ST5 in the Jiangxi population is consistent with what has been reported previously from other parts of China and China’s neighbouring countries, such as Korea, Japan, and Vietnam. The remaining three STs (ST139, ST186, and ST359) were geographically unique, had been reported so far only from Africa, Shanghai and Sichuan Province in China, respectively.

### Phylogenetic Analysis

**Figure 1:**
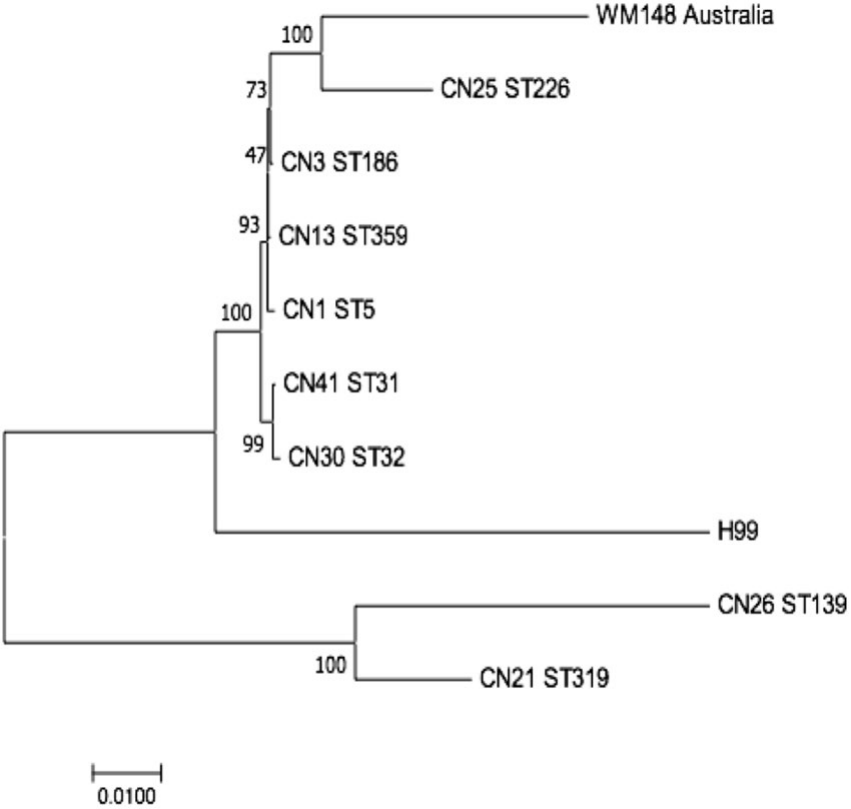
Phylogenetic tree constructed using the Neighbour-joining method, based on the concatenated sequences at seven MLST loci. Only one representative isolate of each ST from Jiangxi Province is shown here. Two reference strains (WM148 and H99) of VNI are included for comparisons.

To further reveal the relationships among the isolates and genotypes, we conducted a Neighbour-joining analysis of the concatenated gene sequences at the seven MLST loci (Fig. 1). Here, only one representative strain of each of the eight sequence types was included in this analysis to allow better visualization. Two reference strains H99 and WM148, both of the VNI molecular type group, were also included (Fig. 1). Our analysis showed that ST5, ST186, and ST359 were genetically very similar, differ from each other by one to a few nucleotides at one (*GPD1*) of the seven sequenced loci (Fig. 1 and Table 2). Similarly, ST31 and ST32 were very close to each other, differed from each other by a few nucleotides at the *PLB1* locus. Overall, these five STs formed a tight cluster with each other. In contrast, the other three STs (ST139, ST226, and ST319) were more distantly related to each other and to those five STs described above based on the concatenated gene sequences at these seven loci.

### Relationships Among Geographic Populations of *C. neoformans* in China

The multilocus genotypes of all isolates from China in the Cryptococcus MLST database were retrieved. A total of 385 isolates from 27 provinces/municipalities in China have been deposited in the database, including the 86 isolates from Jiangxi Province in our study. Among the 27 geographic populations, 12 had isolates of less than five each (most of these 12 populations had only 1–2 isolates each!) and these populations were excluded from our population genetic comparisons. The remaining 15 populations included a total of 364 isolates (Table 4). Our analyses revealed that overall, geographic separation contributed significantly to the total observed genetic variations of the Chinse population of *C. neoformans*. Specifically, AMOVA result showed that about 65% of the observed genetic variation were due to geographic separation while 35% was found within geographic populations (P < 0.001). Among the seven loci, five (*GPD1, IGS1, LAC1, SOD1*, and *URA1*) showed significant geographic differentiations while the remaining two (*CAP59* and *PLB1*) showed no significant differentiations (detailed data not shown). Our further analyses identified that the observed genetic differentiations were mostly due to the genetic uniqueness of the population from Beijing (Table 4). Of the remaining 91(14 × 13/2) pairwise comparisons, only the Jiangxi-Sichuan populations showed statistically significant genetic differentiation (Table 4).

**Table 4:** Evidence for genetic differentiation among geographic populations of *C. neoformans* in China.

	BJ (112)	JX (86)	GD (31)	SH (22)	SC (19)	HN (15)	HK (14)	HEB (13)	ZJ (11)	JS (8)	SD (8)	HUB (7)	LN (7)	AH (6)	SX (5)
Beijing (BJ)		**0.001**	**0.001**	**0.001**	**0.001**	**0.001**	**0.001**	**0.001**	**0.001**	**0.001**	**0.001**	**0.001**	**0.001**	**0.001**	0.002
Jiangxi (JX)	**0.426**		0.379	0.225	**0.012**	0.334	0.118	0.176	0.259	0.336	0.355	0.344	0.331	0.324	0.295
Guangdong (GD)	**0.382**	0.000		0.422	0.059	0.359	0.101	0.429	0.310	0.243	0.313	0.266	0.251	0.212	0.182
Shanghai (SH)	**0.289**	0.009	0.000		0.273	0.390	0.383	0.304	0.327	0.354	0.310	0.346	0.363	0.313	0.263
Sichuan (SC)	**0.195**	**0.093**	0.081	0.000		0.360	0.347	0.196	0.270	0.292	0.127	0.369	0.139	0.450	0.516
Henan (HN)	**0.337**	0.000	0.000	0.000	0.021		0.222	0.525	0.161	0.332	0.363	0.294	0.305	0.270	0.249
Hongkong (HK)	**0.323**	0.031	0.041	0.005	0.026	0.000		0.503	0.489	0.507	0.284	0.377	0.407	0.139	0.063
Hebei (HEB)	**0.369**	0.019	0.002	0.008	0.069	0.000	0.007		0.466	0.397	0.399	0.321	0.333	0.312	0.282
Zhejiang (ZJ)	**0.379**	0.000	0.000	0.007	0.085	0.000	0.015	0.000		0.421	0.711	0.487	0.636	0.573	0.313
Jiangsu (JS)	**0.380**	0.000	0.000	0.000	0.080	0.000	0.009	0.000	0.000		1.000	0.485	0.478	0.440	0.001
Shandong (SD)	**0.381**	0.000	0.000	0.011	0.083	0.000	0.019	0.000	0.006	0.000		0.720	0.738	0.692	0.406
Hubei (HUB)	**0.356**	0.000	0.000	0.000	0.043	0.000	0.000	0.000	0.000	0.020	0.001		1.000	0.740	0.422
Liaoning (LN)	**0.377**	0.000	0.000	0.006	0.074	0.000	0.000	0.000	0.013	0.020	0.001	0.000		0.728	0.423
Anhui (AH)	**0.313**	0.000	0.000	0.000	0.000	0.000	0.000	0.000	0.026	0.051	0.007	0.002	0.002		0.473
Shanxi (SX)	**0.358**	0.000	0.000	0.000	0.027	0.000	0.000	0.000	0.000	0.000	0.000	0.000	0.000	0.000	

The pairwise population *F*_*ST*_ values are shown below diagonal. The probability of the observed *F*_*ST*_ values being statistically significant is shown above diagonal, with a P value of <0.05 rejecting the null hypothesis that the two compared populations are genetically similar to each other.

The abbreviations on the top row are the same as those in the left column. Numbers in parenthesis of the top row are the numbers of isolates from each of the geographic populations.

### Antifungal Susceptibility

The antifungal drug susceptibility results are presented in Table 4. Our comparisons with recommended resistance breakpoints for these drugs indicated that all 86 cryptococcal isolates were susceptible to 5FC, AMB, FCA, ITR, and VRC. Even though no drug resistant cryptococcal isolates were found among these 86 isolates, there are several noteworthy features. First, variations in MIC values were found for all five tested drugs, with as high as 4-fold differences for itraconazole and 8-fold differences for fluconazole and voriconazole. Second, the differences in MICs were not associated with sequence types. For example, strains in ST5 had a range of MIC values similar to those observed in the overall population. Similarly, the MICs of other seven STs were within the range shown by strains of ST5. Third, strains with relatively high MIC values were broadly distributed. For example, five strains with fluconazole MICs of 8 µg/ml were found in four cities/counties (Shangrao, Leping, Yichun, and Nanchang) (Tables 1 and 5). Fourth, there were significant positive correlations in MIC values among the three triazole drugs in the Jiangxi population of *C. neoformans*. Specifically, the Pearson’s correlation coefficients were 0.781, 0.598, and 0.686 respectively for FCA vs. ITR, FCA vs. VRC, and ITR vs. VRC (p values all smaller than 0.001). Finally, despite not being called drug resistant, among the 86 isolates, one (CN29, ST5) showed consistently high MIC values for all five drugs.

**Table 5:** Susceptibilities of the 86*C. neoformans* isolates from Jiangxi Province against five common antifungal drugs.

Isolate	Location	ST^1^	5FC^2^	AMB^3^	FCA^4^	ITR^5^	VRC^6^
CN6	Dexing	5	<4	<0.5	1	0.125	0.06
CN9	Dexing	5	<4	<0.5	2	0.125	0.06
CN1	Fengcheng	5	<4	<0.5	2	0.25	0.125
CN12	Fuzhou	5	<4	<0.5	2	0.25	0.125
CN2	Fuzhou	5	<4	<0.5	4	0.25	0.25
CN26	Fuzhou	139	<4	<0.5	2	0.25	0.125
CN45	Fuzhou	5	<4	<0.5	4	0.25	0.125
CN51	Fuzhou	5	<4	<0.5	2	0.25	0.25
CN56	Fuzhou	5	<4	<0.5	4	0.25	0.25
CN69	Fuzhou	5	<4	<0.5	2	0.125	0.06
CN8	Fuzhou	5	<4	<0.5	2	0.125	0.125
CN76	Fuzhou	359	<4	<0.5	1	0.125	0.06
CN28	Ganzhou	5	<4	<0.5	2	0.125	0.06
CN41	Ganzhou	31	<4	<0.5	4	0.25	0.125
CN63	Ganzhou	5	<4	<0.5	2	0.25	0.25
CN88	Ganzhou	5	<4	<0.5	1	0.125	0.06
CN92	Gaozhou	5	<4	<0.5	1	0.125	0.125
CN37	Gaoan	5	<4	<0.5	2	0.125	0.06
CN14	Ji’an	5	<4	<0.5	4	0.25	0.125
CN21	Ji’an	319	<4	<0.5	4	0.125	0.25
CN49	Ji’an	5	<4	<0.5	2	0.25	0.125
CN50	Ji’an	5	<4	<0.5	4	0.25	0.125
CN53	Ji’an	5	<4	<0.5	4	0.25	0.125
CN61	Ji’an	5	<4	<0.5	2	0.25	0.25
CN73	Ji’an	5	<4	<0.5	2	0.25	0.125
CN74	Ji’an	5	<4	<0.5	2	0.125	0.125
CN79	Ji’an	5	<4	<0.5	1	0.125	0.06
CN94	Ji’an	359	<4	<0.5	2	0.125	0.06
CN31	Jiujiang	5	<4	<0.5	4	0.25	0.125
CN36	JiuJiang	5	<4	<0.5	4	0.25	0.25
CN48	Jiujiang	5	<4	<0.5	4	0.25	0.125
CN64	Jiujiang	5	<4	<0.5	2	0.125	0.125
CN65	Jiujiang	5	<4	<0.5	2	0.25	0.25
CN68	Jiujiang	5	<4	<0.5	4	0.25	0.25
CN86	Jiujiang	5	<4	<0.5	1	0.125	0.125
CN87	Jiujiang	5	<4	<0.5	4	0.125	0.25
CN10	Leping	5	<4	<0.5	2	0.25	0.125
CN19	Leping	5	<4	<0.5	8	0.5	0.25
CN27	Leping	5	4	0.5	2	0.25	0.06
CN3	Leping	186	<4	<0.5	2	0.25	0.125
CN44	Leping	5	<4	<0.5	2	0.125	0.06
CN11	Nanchang	5	<4	1	4	0.25	0.125
CN13	Nanchang	359	<4	<0.5	2	0.25	0.125
CN16	Nanchang	5	<4	<0.5	2	0.25	0.125
CN18	Nanchang	5	<4	<0.5	8	0.5	0.5
CN22	Nanchang	5	<4	<0.5	2	0.125	0.06
CN23	Nanchang	5	<4	0.5	2	0.25	0.125
CN24	Nanchang	5	<4	<0.5	4	0.25	0.125
CN34	Nanchang	5	<4	<0.5	4	0.125	0.125
CN35	Nanchang	5	<4	<0.5	2	0.25	0.25
CN39	Nanchang	5	<4	<0.5	1	0.125	0.06
CN57	Nanchang	5	<4	<0.5	2	0.125	0.06
CN58	Nanchang	5	<4	<0.5	2	0.25	0.125
CN7	Nanchang	5	<4	<0.5	2	0.125	0.125
CN71	Nanchang	5	<4	<0.5	2	0.25	0.06
CN81	Nanchang	5	<4	<0.5	1	0.25	0.125
CN85	Nanchang	359	<4	<0.5	2	0.125	0.125
CN93	Nanchang	5	<4	<0.5	2	0.125	0.06
CN46	Pingxiang	5	<4	<0.5	2	0.125	0.06
CN15	Shangrao	5	<4	1	4	0.125	0.06
CN29	Shangrao	5	4	1	8	0.5	0.25
CN30	Shangrao	32	<4	0.5	4	0.25	0.125
CN32	Shangrao	5	<4	<0.5	4	0.25	0.125
CN33	Shangrao	5	<4	<0.5	2	0.125	0.06
CN4	Shangrao	5	4	<0.5	1	0.125	0.06
CN40	Shangrao	5	<4	<0.5	2	0.125	0.06
CN42	Shangrao	5	4	<0.5	4	0.25	0.125
CN52	Shangrao	5	<4	<0.5	2	0.25	0.125
CN54	Shangrao	5	<4	<0.5	4	0.25	0.25
CN67	Shangrao	5	<4	<0.5	2	0.25	0.25
CN70	Shangrao	5	<4	<0.5	2	0.125	0.06
CN75	Shangrao	5	<4	<0.5	4	0.25	0.125
CN80	Shangrao	5	<4	<0.5	1	0.125	0.06
CN84	Shangrao	5	<4	<0.5	2	0.125	0.125
CN66	Xinyu	5	<4	<0.5	4	0.25	0.25
CN91	Xinyu	5	<4	<0.5	2	0.125	0.06
CN17	Yichun	5	<4	<0.5	8	0.5	0.25
CN25	Yichun	226	<4	<0.5	2	0.125	0.06
CN38	Yichun	5	<4	<0.5	2	0.25	0.125
CN43	Yichun	5	<4	<0.5	8	0.5	0.25
CN60	Yichun	5	<4	<0.5	4	0.25	0.25
CN82	Yichun	186	<4	<0.5	2	0.125	0.06
CN20	Yingtan	5	<4	1	4	0.125	0.125
CN55	Yingtan	5	<4	<0.5	4	0.25	0.25
CN83	Yingtan	186	<4	<0.5	4	0.25	0.125
CN90	Yingtan	186	<4	<0.5	1	0.125	0.125

^1^ST: sequence type as determined based on the combined sequences at the seven loci.

^2^5FC: 5-Flucytocine.

^3^AMB: Amphotericin B.

^4^FCA: Fluconazole.

^5^ITR: Itriconazole.

^6^VRC: Voriconazole.

## Discussions

In this study, we analyzed the genotypes and drug susceptibility profiles of 86 isolates obtained from across Jiangxi Province in China. Our analyses identified eight multilocus sequence types, with five of which represented by only one isolate each. Of the eight STs found in our sample from Jiangxi, six have been reported from other geographic regions while two were novel, identified so far only in Jiangxi Province. The dominant sequence type in Jiangxi, ST5, is a broadly distributed genotype and has been commonly found in other parts of China as well as in the Far East. These eight genotypes show several types of allelic and phylogenetic relationships. Our antifungal drug susceptibility test results showed that none of the 86 strains were resistant to the five tested antifungal drugs. However, some of the strains showed relatively high MIC values. Below we discuss the relevance of our results to earlier studies and the potential implications of these results to the management of cryptococcosis in Jiangxi Province.

Although a considerable amount of information exists on the epidemiology and molecular typing of *C. neoformans* strains in China, there is very little data on cryptococcosis from Jiangxi Province. Studies from the Chinese Mainland, Taiwan, and Hong Kong indicated that the prevalence of cryptococcosis in HIV/AIDS patients ranged from 12.9% to 24.7%, which is significantly lower than that of many other regions in the world^25^. While the total number of HIV-positive patients are not known in Jiangxi Province, HIV-positive patients account for over 40% of the sources of our strains in this study. In contrast, the percentage of isolates from individuals without obvious predisposing risk factors was significantly lower (9/86, 10.5%) than those reported before from other parts of China^26^, but more similar to those from regions outside of China. In Jiangxi Province, cryptococcal infection was more commonly found in middle-aged people, the main group with HIV infections in our samples, than in other age groups. Furthermore, unlike previous studies that found no prominent gender bias in the incidence of cryptococcosis in China^26^, our data showed that the male–female gender ratio was 2.3:1. The ratio in Jiangxi is similar to those reported from Brazil and Europe^27,28^, in which the male to female gender ratio was about 2.9:1.

Similar to observations from other parts of China and other Asian countries, such as Korea, Japan and Thailand^19–32^, our data showed that the 86 clinical isolates of *C. neoformans* from Jiangxi Province had relatively limited amount of genetic variation. All isolates were of the same mating type and the same genotype group VNI. The genotype group VNI is globally the dominant lineage of *C. neoformans* responsible for cryptococcosis^27,28^. A previous study by Fang *et al*.^25^. indicated that serotype A, molecular type VNI, and MATα strains of *C. neoformans* predominate HIV-negative patients in China. Our study suggests this genotype group also predominates the HIV-infected patients in Jiangxi.

To date, seventeen STs of *C. neoformans* var*. grubii* have been identified in China. They include ST5, ST31, ST38, ST53, ST57, ST63, ST93, ST186, ST191, ST194, ST195, ST295, ST296, ST359, and ST360 in Mainland China, while ST4 and ST6 are found in Hong Kong^13,33–35^. In this study, eight STs (i.e. ST5, ST31, ST32, ST139, ST186, ST226, ST319, and ST359) were founded in Jiangxi Province and only four of these eight STs overlap with those reported from other parts of China. This result suggests that there is likely abundant unique genetic diversity of *C. neoformans* in Jiangxi Province. Among the shared STs between Jiangxi and outside of Jiangxi, the majority belonged to ST5, the most common ST in all East Asian countries where epidemiology data are available, including China, Japan, and South Korea^30,36,37^. Interestingly, two other STs, ST31 and ST32, found in Jiangxi were also broadly distributed. According to Khayhan *et al*.^33^, ST139 has so far been found only in Africa. However, its relative frequency in Africa is not known. The geographic distribution patterns of these six shared STs found in Jiangxi suggest that both long- and short- distance dispersals are common in *C. neoformans*. Consistent with this hypothesis, aside from the Beijing population, we found limited evidence for genetic differentiation between most pairs of geographic populations of *C. neoformans* in China. At present, the reason(s) for the genetic distinctiveness of the Beijing population is not known. However, as suggested previously^1,18^, a diversity of factors such as wind, animals such as pigeons, and anthropogenic activities could have contributed to the dispersals of genotypes between Jiangxi Province and other regions both within and outside of China.

In this study, all 86 cryptococcal isolates were susceptible to 5FC, AMB, FCA, ITR, and VRC. Our results suggest that the standard initial therapy for cryptococcosis, AMB combined with 5FC, should still work for patients in Jiangxi Province^38^. However, variations in MICs were observed among the isolates. For each of the drugs, there were isolates showing high MIC values. At present, there was no apparent relationship between MIC to any of the drugs and geographic origins and/or strain genotypes. We would like to note that some of the strains showed high MIC values to multiple drugs. Our results thus call for close monitoring of drug susceptibilities of cryptococcal strains in Jiangxi Province.

In conclusion, our study revealed both shared and divergent genotypes and patterns of cryptococcal epidemiology between Jiangxi Province and other parts of China. Specifically, in both Jiangxi and other parts of China, ST5 was the predominant sequence type. In addition, both unique STs and evidence for long distance dispersals were found among most surveyed regions in China. However, different from previous studies in China, our results identified that most patients in Jiangxi Province with cryptococcosis had underlying risk factors associated with compromised immunity. At present, the mechanism for the predominance of ST5 in East Asian populations is not known. One possibility is that ST5 is more virulent than other sequence types to East Asians. Another possibility is that ST5 was the founder clone in East Asia that has adapted to the local ecological niches. Additional investigations are needed in order to test these possibilities.
